# Sphincter of Oddi Dysfunction and the Formation of Adult Choledochal Cyst Following Cholecystectomy: A Retrospective Cohort Study

**DOI:** 10.1097/MD.0000000000002088

**Published:** 2015-10-30

**Authors:** Hong-Tian Xia, Jing Wang, Tao Yang, Bin Liang, Jian-Ping Zeng, Jia-Hong Dong

**Affiliations:** From the Hospital and Institute of Hepatobiliary Surgery, Chinese PLA General Hospital, Chinese PLA Medical School, Beijing, China.

## Abstract

To determine the causes underlying the formation of adult choledochal cyst.

Anomalous pancreaticobiliary junction is the most widely accepted theory regarding the etiology of choledochal cyst. However, choledochal cysts have been found in patients in the absence of this anomaly. Because the number of adult patients with choledochal cyst is increasing, it is important to address this controversy.

Bile amylase levels in the cysts of 27 patients (8 males and 19 females) who had undergone cholecystectomy were retrospectively evaluated.

The average age of the 27 patients was 45.8 ± 10.1 years and the majority (85.2%) were diagnosed with Todani type I cysts. None of the patients had dilatation of the common bile duct prior to surgery. There were 6 (22.2%) patients with anomalous pancreaticobiliary junction. However, amylase levels did not significantly differ between patients with and without this anomaly (*P* = 0.251). According to bile amylase levels, pancreatobiliary reflux was present in 21 (77.8%) patients. The mean amylase level significantly differed in patients with pancreatobiliary reflux (23,462 ± 11,510 IU/L) and those without (235 ± 103 IU/L) (*P* < 0.001). In patients with pancreatobiliary reflux, only 4 patients had anomalous pancreaticobiliary junction. That is, the majority of patients (17/21, 81%) having pancreatobiliary reflux did not have an anomalous junction of the pancreatic and biliary ducts.

Since the only explanation for pancreatobiliary reflux in patients with a normal pancreaticobiliary junction is sphincter of Oddi dysfunction, we proposed that the formation of adult choledochal cyst is mainly due to sphincter of Oddi dysfunction.

## INTRODUCTION

The etiology of the choledochal cyst (CC) includes both congenital and acquired origins.^1^ The most widely accepted theory regarding the etiology of CCs is that CCs are caused by an anomalous pancreaticobiliary junction (APBJ).^2^ Babbitt et al^3^ suggested that an APBJ allows regurgitation of pancreatic juices into the common bile duct (CBD) resulting in inflammation and deterioration of the bile duct wall leading to dilatation and eventual formation of the CC. However, this theory is questioned because only 50% to 80% of cases of CCs are associated with this anatomic anomaly.^4^ Furthermore, Sai et al^5,6^ have shown that pancreatobiliary reflux can occur in the absence of APBJ (ie, occult pancreatobiliary reflux).

It has been proposed that abnormal contractility of the sphincter of Oddi can explain pancreatobiliary reflux in patients with normal pancreaticobiliary junctions,^7–10^ due to the intraluminal pressure differences between the pancreatic duct and CBD.^11^ Although studies have suggested an association between sphincter of Oddi dysfunction and the formation of CC,^12,13^ there is limited data supporting this phenomenon, especially in adult patients presenting with a CC.^14^ As Polido et al^15^ has indicated, the etiology of adult CC is unclear, although it is most likely multifactorial. As the number of adults presenting with CCs is increasing,^16,17^ it is clinically important to address this controversy over the etiology of CC in an effort to guide their treatment.

The aim of this retrospective study was to determine the cause (or causes) underlying the formation of adult CC. This was accomplished by measuring the bile amylase levels in the CCs of patients who had no biliary ductal dilation prior to cholecystectomy for gallstones, and/or gallbladder polyps.

## SUBJECTS AND METHODS

The study was approved by the Institutional Review Board of our hospital. Due to the retrospective nature of the study, no patient informed consent was required.

The records of patients diagnosed with adult CC, who underwent cholecystectomy at our hospital from January 2000 to December 2012, were reviewed. Radiologic imaging studies used to diagnose adult CCs included computed tomography, magnetic resonance cholangiopancreatography, and ultrasonography, and the diagnosis was confirmed by serologic testing and histopathological findings. Patients with a bile duct diameter more than 10 mm at the time of cholecystectomy were excluded from the study.

Patients with postoperative compensatory dilatation of the CBD, secondary biliary dilatation due to a CBD stone or distal CBD stricture, or endoscopic sphincterotomy-treated patients were also excluded. A total of 27 out of 86 patients who underwent cholecystectomy fulfilled all of the inclusion and exclusion criteria.

**FIGURE 1:**
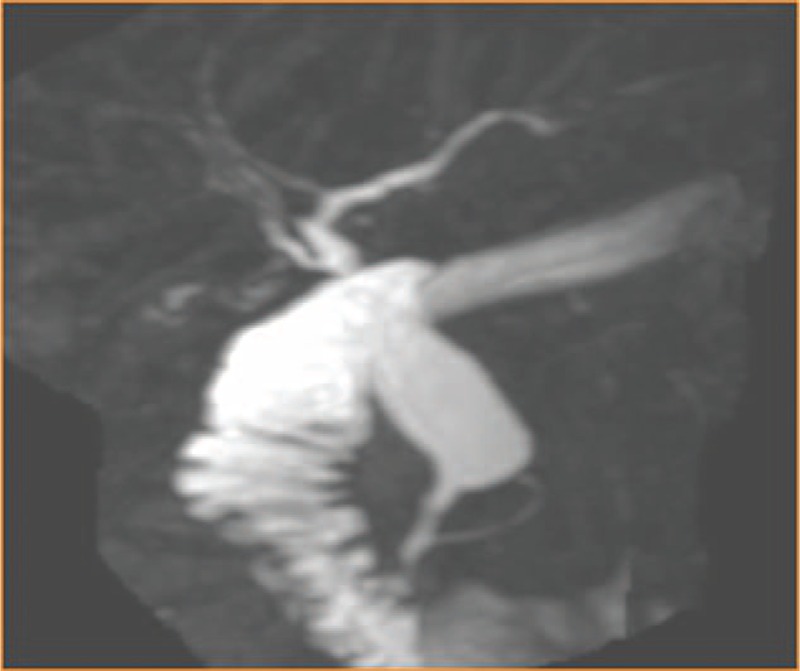
Magnetic resonance cholangiopancreatography image showing anomalous pancreaticobiliary junction (P–B type) and choledochal cyst in a 32-year-old woman.

**FIGURE 2:**
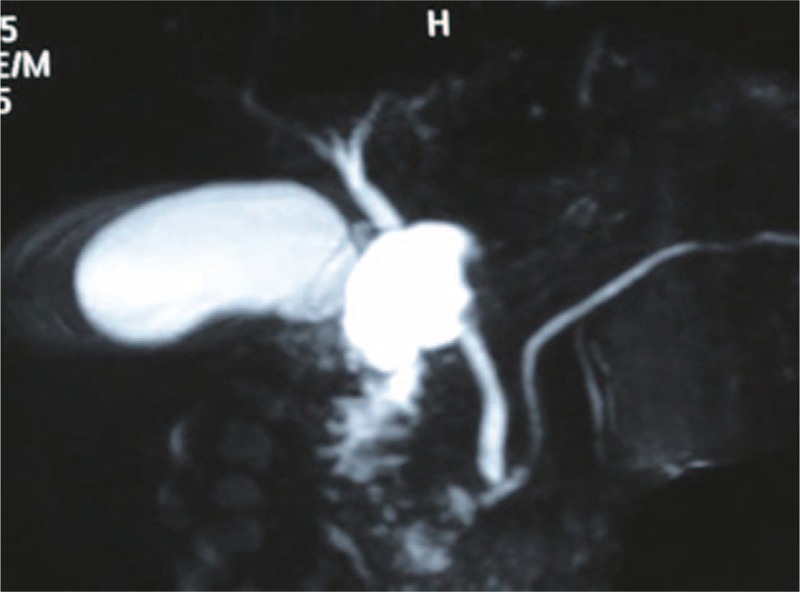
Magnetic resonance cholangiopancreatography image showing anomalous pancreaticobiliary junction (B–P type) in a 52-year-old woman. While an enlarged gallbladder is observed, the patient did not present with choledochal cyst or biliary dilatation.

APBJ was defined as the junction between the pancreatic and bile ducts located outside the duodenal wall with a common channel length >1.5 cm.^18^ This anomaly was further divided into P–B type (where the pancreatic duct joins the CBD; Fig. 1) and B–P type (where the CBD joins the pancreatic duct; Fig. 2).

When the biliary amylase level was >1000 IU/L, pancreatobiliary reflux was considered present.^19^ The amylase level was measured using a colorimetric method with an automatic Roche/Hitachi (cobas c701 module) analyzer (Mannheim, Germany). The normal range of serum amylase using this system is <150 IU/L.

### Statistical Analysis

Descriptive statistics were used to summarize the data. Independent sample *t* tests were used to examine group differences in average amylase levels. Statistical analyses were performed with IBM SPSS Version 20 (SPSS Statistics V20, IBM Corporation, Somers, NY). The statistical significance level was set at a *P* value <0.05.

## RESULTS

The demographic and clinical characteristics of the 27 patients evaluated in this study are presented in Table 1. The average age of the 27 patients (8 males and 19 females) was 45.8 ± 10.1 years, and the majority were diagnosed with Todani type I cysts (85.2%). Of the 27 patients, there were 15 patients with gallstones, 9 patients with gallbladder polyps, and 3 patients with both gallbladder polyps and gallstones. During cholecystectomy, 44.4% of patients had a CBD diameter in the 6 to 8 mm range and 55.6% of patients had a CBD diameter in the 8 to 10 mm range. Following cholecystectomy, over half of the patients had abdominal pain (88.9%), fever (51.9%), and cholangiolithiasis (51.9%). The average duration between cholecystectomy and a definitive diagnosis of CC in the 27 patients was 115.1 ± 35.0 months. During CC excision, the mean CBD diameter was 38.1 ± 10.3 mm.

**TABLE 1:**
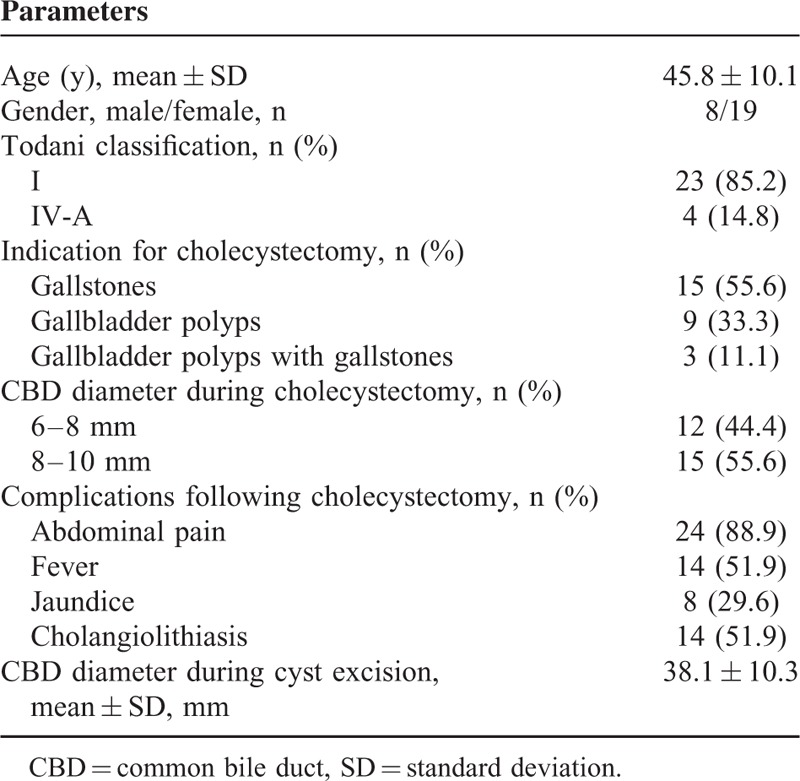
Demographic and Clinical Characteristics of the Patients (n = 27)

**FIGURE 3:**
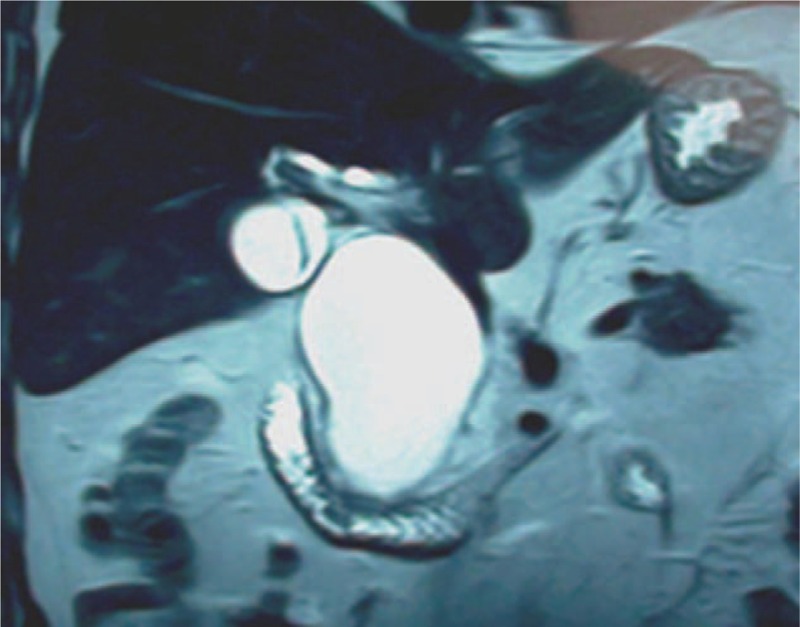
Magnetic resonance cholangiopancreatography image showing the normal type of pancreaticobiliary duct junction in a 38-year-old man presenting with adult choledochal cyst, which was confirmed at surgery.

**FIGURE 4:**
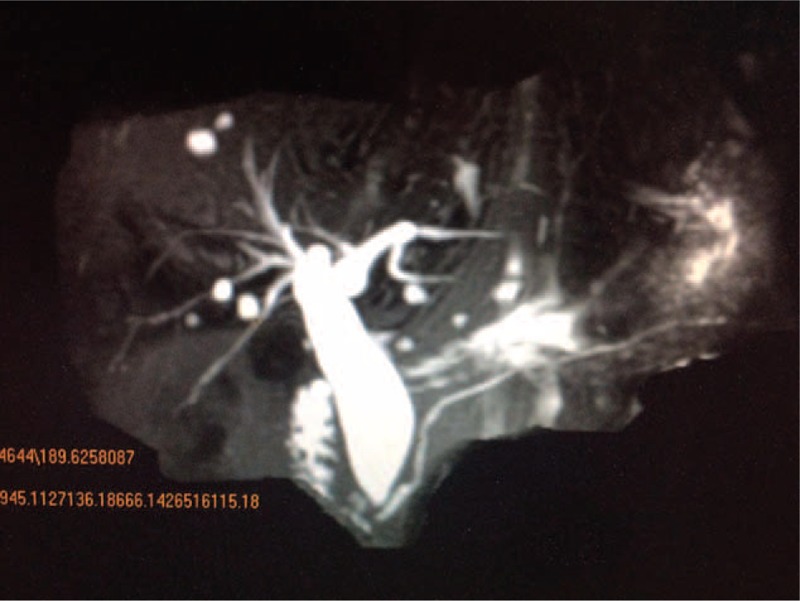
Magnetic resonance cholangiopancreatography image of a 42-year-old woman presenting with a choledochal cyst 68 months after receiving a cholecystectomy.

As shown in Table 2, APBJ was found in 6 (22.2%) patients. However, amylase levels did not significantly differ between patients with APBJ and those without APBJ (*P* = 0.251). According to bile amylase levels measured in the CC, pancreatobiliary reflux was present in 21 (77.8%) patients. The mean amylase level significantly differed between patients with pancreatobiliary reflux (23,462 ± 11,510 IU/L) and those without pancreatobiliary reflux (235 ± 103 IU/L; *P* < 0.001). Of note, in patients with pancreatobiliary reflux, only 4 patients presented with APBJ. That is, occult pancreatobiliary reflux accounted for the majority of patients (17/21, 81%) who presented with reflux of pancreatic juice into the biliary tract. Figure 3 shows a normal pancreaticobiliary duct junction in a 38-year-old man presenting with adult CC which was confirmed at surgery. Figure 4 shows a 42-year-old woman who presented with a CC 68 months after receiving a cholecystectomy.

**TABLE 2:**
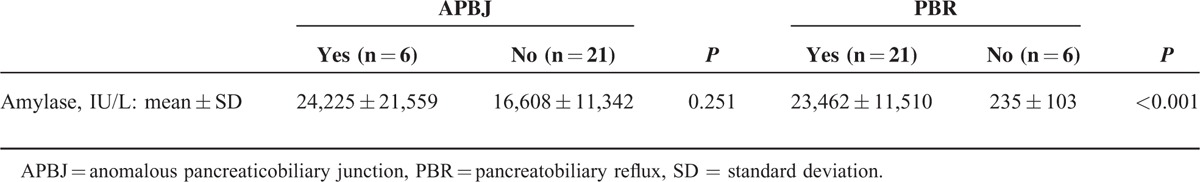
Comparison of Amylase Levels

## DISCUSSION

Our results showed that, based on bile amylase levels, pancreatobiliary reflux was present in 21 (77.8%) patients with CCs and of those 21 patients, only 4 had APBJs. That is, occult pancreatobiliary reflux accounted for the majority of patients (17/21, 81%) who presented with reflux of pancreatic juice into the biliary tract and it appears that pancreatobiliary reflux was responsible for formation of CCs.

The present study demonstrated that pancreatobiliary reflux cannot be fully explained by an APBJ as APBJ was found in only 4 patients with pancreatobiliary reflux and amylase levels did not significantly differ between patients with APBJ and those without APBJ. In other words, the cause of pancreatobiliary reflux cannot be fully explained by the theory of an APBJ. In addition to APBJs, other mechanisms can lead to the reflux of pancreatic enzymes into the biliary tract that may trigger the formation of adult CC. Due to bile duct dilatation after cholecystectomy, an association between gallbladder resection and the formation of adult CC is also possible.^20^

The CBD and main pancreatic duct open into the duodenum either at separate points or via a common channel.^21^ At the lower end of the bile and pancreatic ducts, the sphincter of Oddi regulates the flow of bile and pancreatic juice into the duodenum.^22^ When the common channel exceeds 15 mm in length (ie, an anomalous junction), the sphincter of Oddi is inoperative. As a consequence, the pancreatic enzymes flow freely into the biliary system causing pancreatobiliary reflux.^21^ Although it is well known that pancreatobiliary reflux is frequently associated with the formation of CC,^23^ patients with an anomalous junction of pancreatic and biliary ducts may not present with CC.^24,25^ It is also possible to observe CC in patients without an anomalous junction of the pancreatic and biliary ducts.^24^ In the present study, we found over 80% of the patients with pancreatobiliary reflux had an anatomically normal pancreaticobiliary junction. This phenomenon of pancreatobiliary reflux found in patients with a normal pancreaticobiliary junction was defined as occult pancreatobiliary reflux.^26^

It has been suggested that sphincter of Oddi dysfunction is the only plausible explanation for pancreatobiliary reflux in patients with a normal pancreaticobiliary junction.^7,27,28^ Ponce et al^13^ utilized endoscopic manometry to investigate the motility of the sphincter of Oddi and observed that patients with biliary cystic dilatation and those with CC both showed an elevated basal pressure but the frequency of phasic contractions of the sphincter of Oddi was abnormal only in patients with CC. Craig et al^12^ provided manometric evidence of an elevated basal sphincter pressure of 59 mm Hg in an adult patient found to have CC but who did not show an APBJ by endoscopic retrograde cholangiopancreatography (ERCP) and suggested an etiological role of an abnormal sphincter of Oddi in the formation of CC.

The sphincter of Oddi is a cylindrically shaped smooth muscle located where the distal CBD and the main pancreatic duct meet at the entrance to the duodenum.^14^ The role of the sphincter of Oddi is to regulate the flow of bile and pancreatic enzymes into the duodenum by antegrade phasic contractions.^27^ Therefore, sphincter of Oddi dysfunction is a term referring to the malfunction of sphincter of Oddi contractility,^14^ which results in retrograde contractions allowing the regurgitation of pancreatic enzymes into the biliary tree. In the present study, we found elevated biliary amylase levels, a biochemical marker of pancreatobiliary reflux,^7^ in 21 (77.8%) patients with CCs. In the 21 patients presenting with pancreatobiliary reflux, APBJ was found in only 4 patients. That is, occult pancreatobiliary reflux accounted for the majority of patients with the reflux of pancreatic juice into the biliary tract. Since the only reasonable explanation for occult pancreatobiliary reflux in patients with a normal pancreaticobiliary duct junction is sphincter of Oddi dysfunction,^7,27,28^ we postulated that, apart from an APBJ, the formation of adult CC is likely due to sphincter of Oddi dysfunction.

According to our results, no patient had obvious dilatation of the CBD or intrahepatic bile ducts before cholecystectomy, but developed cystic dilatation of the bile ducts after cholecystectomy, indicating a causal relationship between cholecystectomy and the formation of adult CC. This specific association requires further elucidation.

The primary functions of the gallbladder are to store and concentrate bile, regulate bile release according to the body's physiological needs, and together with the sphincter of Oddi, regulate bile duct pressure through contraction and relaxation of the gallbladder.^29^ More specifically, the gallbladder motor activity and the sphincter of Oddi motility are integrated with the secretion of gut hormones such as the cholecystokinin (CCK).^30^ For example, during the digestive period, CCK leads to the contraction of the gallbladder and the relaxation of the sphincter of Oddi, which enables the bile to discharge into the duodenum. That is, the contraction and relaxation of the gallbladder and the sphincter of Oddi are closely coordinated with the digestive system by neurohormonal mechanisms during the fasting state and the digestive periods.

When the body is in the fasting state, the sphincter of Oddi closes, resulting in increased bile duct pressure forcing bile to flow into the gallbladder while the pressure within the bile ducts decreases.^29^ Physiological studies have shown that the sphincter of Oddi generates prominent phasic contractions (100–150 mm Hg) which are orientated in a primarily antegrade direction and contract at a frequency of 2 to 6 per minute.^31,32^ Bile flows mainly by a passive mechanism in between these phasic contractions.^31,33^ The frequency of contractions varies during fasting and shows an increase just before phase III duodenal activity.^31,33,34^ This complex motility is orchestrated by a neurohormonal interplay, whose precise details have yet to be determined.^31^

After eating a fatty meal, through neurohumoral regulation, the sphincter of Oddi relaxes as the amplitude of the contractions decrease facilitating increased flow of bile from the bile duct into the duodenum by passive flow between contractions.^31,33^ Biliary tract pressure then drops and in the interim, the gallbladder contracts and a large amount of bile juice is released into the duodenum. The above analysis indicates that the biliary system (composed of the biliary tree, gallbladder, ampulla of Vater [located within the major duodenal papilla which is the opening of the pancreatic duct into the duodenum], sphincter of Oddi, and a complex neurohormonal mechanism) works as a unit. Any defect in any part of that system can destroy its balance and affect its normal functioning.

Once the gallbladder is removed, the biliary system loses the regulatory function provided by the gallbladder, leading to abnormal bile storage and excretion as well as abnormal pressure regulation within the bile duct. When the stomach is empty, the contraction of the sphincter of Oddi within the ampulla of Vater contracts (closes) and bile duct pressure elevates. Since the regulatory function of the gallbladder has been removed, secondary biliary ductal dilatation will occur to relieve the bile duct pressure, which is the cause of secondary biliary ductal dilation after cholecystectomy. During eating, the papillary sphincter is relaxed through neurohumoral regulation, the bile drains into the duodenum and the bile excretion is increased through the contraction of smooth muscle within the bile duct wall; the balance of the bile duct pressure can, therefore, be maintained through the contraction of the smooth muscle within the bile duct wall. Thus, although gallbladder resection leads to secondary biliary ductal dilation, the dilation is not serious due to good functioning of the smooth muscle within the walls of the biliary ducts and normal functioning of the sphincter of Oddi. At this time, the biliary system is in compensatory balance after cholecystectomy, and normal biliary function is maintained.

Several conditions are responsible for the dysfunction of the sphincter of Oddi that results in pancreatobiliary reflux.^7,35,36^ From clinical observations, patients with pancreatobiliary reflux do not necessarily have cystic dilation of the bile duct. When the degree of pancreatobiliary reflux is relatively mild, the amount of refluxed pancreatic juice is small. A small amount of pancreatic juice that flows into the bile ducts has a relatively weak erosive effect on the bile ducts and also has a relatively weak destructive effect on the smooth muscle within the bile duct wall as it is diluted by large amounts of bile stored in the gallbladder. At this time, the erosive effect of the pancreatic juice is mainly exerted on the gallbladder that stores the bile, with the clinical symptoms of enlarged gallbladder volume and decreased gallbladder contractility and is responsible for the initial clinical manifestations of pancreaticobiliary reflux, that is, cholecystitis. At this time, the biliary system can still maintain relatively normal physiological function, so some patients with a relatively mild degree of pancreatobiliary reflux do not develop CCs. However, if these patients have their gallbladder removed, the balance is broken, the erosive effect of regurgitated pancreatic juice transfers from the gallbladder to the bile duct and the long-term erosive effect of pancreatic juice causes a gradual loss of bile duct smooth muscle.^5,7,37^ Thus, the role played by the bile duct in maintaining bile duct pressure through its smooth muscle contraction is also gradually lost. Under these conditions, the bile duct pressure gradually elevates, and the bile duct gradually dilates leading to the formation of CCs. Therefore, we advocate that the structure and function of the biliary system, as a whole, be maintained as much as possible.

With the popularity of laparoscopic techniques, the number of cholecystectomies has increased significantly. At our center, for example, before 2000, cholecystectomy was primarily performed by conventional laparotomy with an average of 150 to 200 surgical cases per year. Since 2000, cholecystectomy has been performed primarily by laparoscopic technique and the number of surgical cases has increased. At present, approximately 1000 cholecystectomies are performed per year at our center. The clinical data also show that the number of adult cases of CC has increased every year since 2000, developing from approximately 10 cases per year before 2000 to 69 cases per year in 2012. This growth trend is very significant. Among the adult cases with CC treated in recent years, the number of patients who had received cholecystectomy has also increased. Therefore, a certain relationship exists between cholecystectomy and the formation of adult CC.

The most serious consequence of CC formation is the development of hepatobiliary cancer. The bile and pancreatic juice within the CC from pancreatobiliary reflux not only causes cystic dilatation of the bile duct but also causes prolonged erosion of the bile duct system by activated pancreatic trypsin.^38,39^ The addition of long-term cholestasis and inflammatory stimulation secondary to cholangitis results in carcinogenesis within the cystic ducts.^40,41^

Of all hepatobiliary cancers, cholangiocarcinoma (CCC) is the most frequent histological type encountered.^42,43^ CCs are highly prevalent in Asia, especially in China.^40,44^ Once CCC develops, it is highly malignant with poor outcome.^40^ Therefore, the primary purpose of CC surgery is to interrupt the natural course of CCC and prevent its development through early surgical cyst removal.

The pathological change associated with CCC formation within CCs also provides conditions for the formation of cholangiolithiasis. When cholangiolithiasis occurs recurrently after cholecystectomy, the possibility of pancreatobiliary reflux must be considered. It can be seen from our analysis that the biliary system will be affected if any aspect becomes abnormal. During clinical practice, therefore, a complete and coordinated biliary system should be preserved as much as possible. Once the balance of biliary function is destroyed, it will be very difficult for the body to maintain normal biliary function. We propose that the clinicians should try their best to preserve the structure and function of the biliary system during their clinical practice.

This study had several limitations including its retrospective nature and small sample size (27 patients). In addition, the pancreatic juice could not be detected in the bile of 6 (22.2%) patients. We were unable to measure the bile duct pressure in patients with CCs undergoing surgery as the contractile function of the sphincter of Oddi was affected by the anesthesia. Therefore, future prospective studies involving larger cohorts of patients with CC and bile duct pressure measurements are needed to confirm our results. In addition, assessment of sphincter of Oddi structure and function is also necessary as it may aid in understanding the mechanism underlying the formation of CCs. The present study was limited as it was only able to secondarily infer abnormal structure and function of the sphincter of Oddi based on the detection of pancreatic juice (as evidenced by elevated amylase levels) within the bile. Since imaging studies may aid in the evaluation of abnormal structure and function of the sphincter of Oddi, they should also be included in future research studies.

In conclusion, pancreatobiliary reflux appears to be the primary cause of CC formation. Pancreatobiliary reflux can be caused by either APBJ or sphincter of Oddi dysfunction (occult pancreatobiliary reflux). Occult pancreatobiliary reflux appears responsible for the majority of our adult cases of CC. Thus, our findings suggest that the formation of adult CC is primarily due to sphincter of Oddi dysfunction.
